# Chlorhexidine hexametaphosphate nanoparticles as a novel antimicrobial coating for dental implants

**DOI:** 10.1007/s10856-015-5532-1

**Published:** 2015-06-30

**Authors:** Natalie J. Wood, Howard F. Jenkinson, Sean A. Davis, Stephen Mann, Dominic J. O’Sullivan, Michele E. Barbour

**Affiliations:** Oral Nanoscience, School of Oral & Dental Sciences, University of Bristol, Bristol, UK; Centre for Organized Matter Chemistry, School of Chemistry, University of Bristol, Bristol, UK; Bristol Centre for Functional Nanomaterials, University of Bristol, Bristol, UK; Oral Microbiology, School of Oral & Dental Sciences, University of Bristol, Bristol, UK

## Abstract

Dental implants are an increasingly popular solution to missing teeth. Implants are prone to colonisation by pathogenic oral bacteria which can lead to inflammation, destruction of bone and ultimately implant failure. The aim of this study was to investigate the use of chlorhexidine (CHX) hexametaphosphate (HMP) nanoparticles (NPs) with a total CHX concentration equivalent to 5 mM as a coating for dental implants. The CHX HMP NPs had mean diameter 49 nm and composition was confirmed showing presence of both chlorine and phosphorus. The NPs formed micrometer-sized aggregated surface deposits on commercially pure grade II titanium substrates following immersion–coating for 30 s. When CHX HMP NP-coated titanium specimens were immersed in deionised water, sustained release of soluble CHX was observed, both in the absence and presence of a salivary pellicle, for the duration of the study (99 days) without reaching a plateau. Control specimens exposed to a solution of aqueous 25 µM CHX (equivalent to the residual aqueous CHX present with the NPs) did not exhibit CHX release. CHX HMP NP-coated surfaces exhibited antimicrobial efficacy against oral primary colonising bacterium *Streptococcus gordonii* within 8 h. The antimicrobial efficacy was greater in the presence of an acquired pellicle which is postulated to be due to retention of soluble CHX by the pellicle.

## Introduction

Titanium is the major component of most dental implant systems, since it exhibits excellent biocompatibility and supports osseointegration. The rate of osseointegration is affected by surface roughness; an implant with moderate roughness of 1–2 µm is thought to be optimal [1]. With rougher implant surfaces comes an increased susceptibility to bacterial adhesion, at least in vitro [2–4], and this has been attributed to increased protection from shear forces [4]. Drawing inference from such in vitro data, it has been proposed that rough implant surfaces may exhibit a greater propensity for implant-associated infections in vivo [5]. Peri-implant mucositis, the most common form of infection, occurs in approximately 80 % of subjects [6], resulting in reversible inflammation of peri-implant soft tissues. The more severe peri-implantitis occurs in about 28–56 % of subjects [6] and, as well as soft tissue inflammation, results in loss of the supporting bone. Treatment of peri-implantitis has unpredictable outcomes and control of plaque in the mouth surrounding the implant is essential [7].

Upon exposure to the oral cavity, implants, like all materials, are rapidly coated with a thin proteinaceous film known as the salivary pellicle. This is composed primarily of salivary glycoproteins and mediates adhesion of oral primary colonisers [8]; the most common such species found on dental implants are the streptococci [9]. Once adhered, the bacteria proliferate and excrete an extracellular polysaccharide matrix which protects the developing microcolony. Secondary colonising bacteria then adhere to the primary colonisers resulting in biofilm formation. The microbiota found in infected implant sites have similarities to those seen in periodontitis [10], although with somewhat less diversity [11].

Chlorhexidine (CHX) is a broad spectrum antimicrobial and antifungal agent belonging to the biguanide class of drugs. It is used extensively in medicine and dentistry, as the soluble digluconate salt, for a wide range of applications including mouthrinses, eye drops and as a pre-surgical topical cleansing agent. Its non-specific mode of action is associated with rupture of the bacterial cell membrane resulting in leakage of intracellular components [12]. CHX adsorbs to titanium oxide surfaces, and the resultant CHX-coated surface can reduce growth of *Streptococcus gordonii* [13], but the CHX is rapidly depleted providing only a short-term antimicrobial effect [14].

Antimicrobial nanoparticles (NPs) offer a method for imparting antimicrobial properties to implant surfaces. One advantage of NPs as a coating for dental implants when not embedded in a film but applied directly to the titanium is that, with careful control of the doping and distribution, their small size provides the opportunity to leave the majority of the titanium surface uncoated and thus available for osteoblast colonisation and subsequent osseointegration. Elemental silver is used as an antimicrobial agent in many fields of medicine, and silver NPs embedded in various film coatings have been applied to titanium implant surfaces for the purpose of conferring antimicrobial activity [15–19]. However, cytotoxic effects of silver NPs have been reported and thus safety concerns persist [20, 21]. Chlorhexidine–hexametaphosphate (CHX–HMP) NPs have recently been reported and act as a slow release device for soluble CHX [22]. The research question was: do CHX–HMP NPs have potential as an antimicrobial coating for titanium dental implants. The hypothesis to be tested was: CHX–HMP NPs have no antimicrobial efficacy when used as a coating for titanium.

## Materials and methods

### Nanoparticle synthesis, specimen preparation and characterisation

100 mL 10 mM aqueous sodium HMP (Sigma-Aldrich Company Ltd, Dorset, UK) was added to 100 mL 10 mM of aqueous CHX digluconate (Sigma-Aldrich Company Ltd, Dorset, UK) under constant stirring and ambient conditions. This resulted in a suspension of CHX–HMP NPs, with a total concentration of 5 mM of both CHX and HMP.

Approximately 20 µL of NP suspension was deposited on a 200 mesh carbon-coated copper/gold grid (Agar Scientific, Essex, UK), left undisturbed for 2 min, then placed on filter paper to dry. Specimens were imaged using transmission electron microscopy (TEM; JEM 1200 EX MKI: Jeol, Welwyn Garden City, UK). In-situ energy-dispersive X-ray spectroscopy (EDX; ISIS 300: Oxford Instruments, Bristol, UK) was used to determine the elemental composition of the NP precipitate.

Square sections (10 × 10 × 1 mm) of grade 2 commercially pure titanium (Ti-TEK Ltd, Sutton Coldfield, UK) were polished using 120 grit silicon carbide paper, followed by 10 min ultrasonication in acetone and 10 min ultrasonication in industrial methylated spirits, before being allowed to dry in air. To coat the titanium specimens with nanoparticles, individual substrates were suspended in 200 mL of the colloidal suspension for 30 s during rapid stirring, followed by 10 s immersion in deionised water, before being blotted to remove excess water and allowed to dry in air. Selected specimens were coated with gold–palladium alloy, using a sputter coater (SC7620, Quorum Technologies, East Grinstead, UK) and imaged using scanning electron microscopy (SEM) (Phenom, Phenom-World, Eindhoven, Netherlands).

### CHX elution from CHX–HMP NP coated titanium

The release of soluble CHX from the CHX–HMP NP coated specimens was recorded as a function of time. The experiments were conducted with and without the application of a salivary pellicle to the NP-coated titanium, to determine whether CHX elution was impeded by the presence of a pellicle. Control groups were titanium substrates treated with deionised water or a 25 µM CHX solution (the residual CHX concentration in the NP suspension) instead of the NP suspension.

Titanium specimens for elution studies were prepared and coated with NPs as described above. Control specimens were immersed in deionised water for 40 s (deionised water controls) or immersed in 25 µM CHX solution for 30 s exposure followed by 10 s in deionised water (aqueous CHX controls).

For those specimens to be coated with a salivary pellicle, stimulated saliva was collected from 5 donors (saliva bank REC reference: 08/H0606/87+5). The saliva was pooled and 0.02 w/v% sodium azide was added to prevent bacterial growth. Titanium specimens were incubated, after coating (with NPs or control treatments), at 37 °C for 2 h in pooled, whole saliva (1 mL/specimen). They were removed and rinsed in deionised water for 5 min (1 mL/specimen) immediately prior to elution experiments.

Specimens were placed in UV-transparent cuvettes (Fisher Scientific, Loughborough, UK), immersed in 3 mL deionised water and sealed with tight-fitting lids with the joint wrapped with Parafilm (Bemis, Londonderry, UK). Specimens were agitated on an orbital shaker (Stuart^®^SSM1; Bibby Scientific Ltd, Stone, UK) at 150 rpm. CHX concentration was measured periodically by recording absorbance at 255 nm using a UV spectrophotometer (U-1900: Hitachi, Tokyo, Japan) and correlating with calibration standards of 5–50 µM CHX [14]. On completion of elution studies the substrates were removed from the cuvettes, blotted to remove excess water and left to dry in air. The surfaces were then imaged using SEM.

### Antimicrobial efficacy against *Streptococcus gordonii*

*Streptococcus gordonii* colonisation of titanium substrates was investigated as a function of NP coating with and without a salivary pellicle. The proliferation of bacteria in the media in the well plates was also determined.

Yeast–nitrogen–phosphate–tryptone (YNPT) medium was prepared by mixing 1× yeast nitrogen base (Appleton Woods, Birmingham, UK), 0.05 % tryptone (BD, Oxford, UK) and phosphate buffer, prepared from 10 mM Na_2_HPO_4_ (Fisher Scientific, Loughborough, UK) and KH_2_PO_4_ (Sigma-Aldrich, Dorset, UK), pH 7.

*Streptococcus gordonii* DL1 cells were grown in screw cap bottles in 10 mL YNTP medium supplemented with 0.5 mL of BHY, prepared from Brain Heart Infusion (Lab M Ltd, Lancashire, UK) and Bacto Yeast Extract (BD, Oxford, UK) and 10 µL of 40 % aqueous glucose solution (Sigma-Aldrich) (YNPTG medium) at 37 °C, overnight. The resulting culture was centrifuged and re-suspended in fresh YNTPG medium, twice. The optical density of the suspension was adjusted to OD_600_ = 0.01 (approximately 5 × 10^6^ cells/mL).

Saliva from human subjects was collected and stored under approval from the National Research Ethics Committee South Central Oxford (# 08/H0606/87+5). Pooled human saliva from at least six adult subjects, who provided written informed consent, was mixed with dithiothreitol to final concentration 2.5 mM, before being centrifuged (12,000*g* for 10 min), and re-suspended at 10 % solution with DI water. The saliva was then filtered through a 0.45 µm filter (Sastedt, Leicester, UK) and stored at −20 °C until required.

NP-coated and uncoated titanium substrates were placed in a 12-well microtitre plate (Greiner Bio-one, Stonehouse, UK), immersed in 1 mL of 10 % saliva and incubated at 4 °C for 2 h. They were then rinsed in 1 mL phosphate buffered saline (PBS) (Sigma-Aldrich Company Ltd, Dorset, UK) for 3 min on a see-saw rocker (Stuart^®^SSL4: Bibby Scientific Ltd, Stone, UK) at 20 rpm, immediately prior to microbiology assays.

Titanium specimens (NP-coated and uncoated control samples, with or without an acquired salivary pellicle) were placed into the wells of 12-well microtitre plates. A 1 mL aliquot of YNTPG growth medium was added, followed by the addition of a further 1 mL of bacterial culture. The specimens were incubated at 37 °C while being agitated at 50 rpm. Specimens were removed at times 0, 8, 24, 48 h and rinsed in fresh YNTPG medium for 3 min on a Stuart see-saw rocker at 20 rpm before analysis. The experiment was performed three times with two specimens for each time point on each occasion, giving a total n = 6 for each measurement time.

After each titanium specimen was removed and rinsed in fresh media, they were placed in 1 mL PBS and agitated using a vortex mixer for 30 s, rested for 3 min, and then vortex-mixed for a further 30 s. The PBS containing released bacteria was then serially diluted by 10, 10^2^, 10^3^ and 10^4^ in fresh PBS; 20 µL aliquots of the initial PBS solution and each dilution were spotted three times onto dry BHYN plates and incubated overnight. Media from the wells were also diluted and spotted onto BHYN agar plates using the same method. The resulting colony forming units (CFUs) were then counted. In order to assess statistical significance, a two-way analysis of variance (ANOVA) in combination with a post hoc Tukey’s range test was performed using SPSS (IBM, Portsmouth, UK). Differences were deemed significant when *P* < 0.05.

### Preparation of specimens for live/dead fluorescent imaging

Substrates were prepared in the same way as described for the growth of the *S. gordonii* on titanium surfaces. Once rinsed in YNTPG medium, substrates were placed in the well of a new microtitre plate before being immersed in fresh YNTPG media containing 3 µL of an equimolar solution of BacLight SYTO^®^ 9 green fluorescent stain and propidium iodide (Live Technologies, Paisley, UK) before a 15 min period of dark incubation. The specimens were then removed from the solution and immediately imaged using fluorescent optical microscopy (Leica DMLB: Leica Microsystems, Milton Keynes, UK). The SYTO 9 stain labelled all bacteria (green) in the population whereas the propidium iodide penetrated only those bacteria with damaged membranes (red).

## Results

### Specimen characterisation

**Fig. 1:**
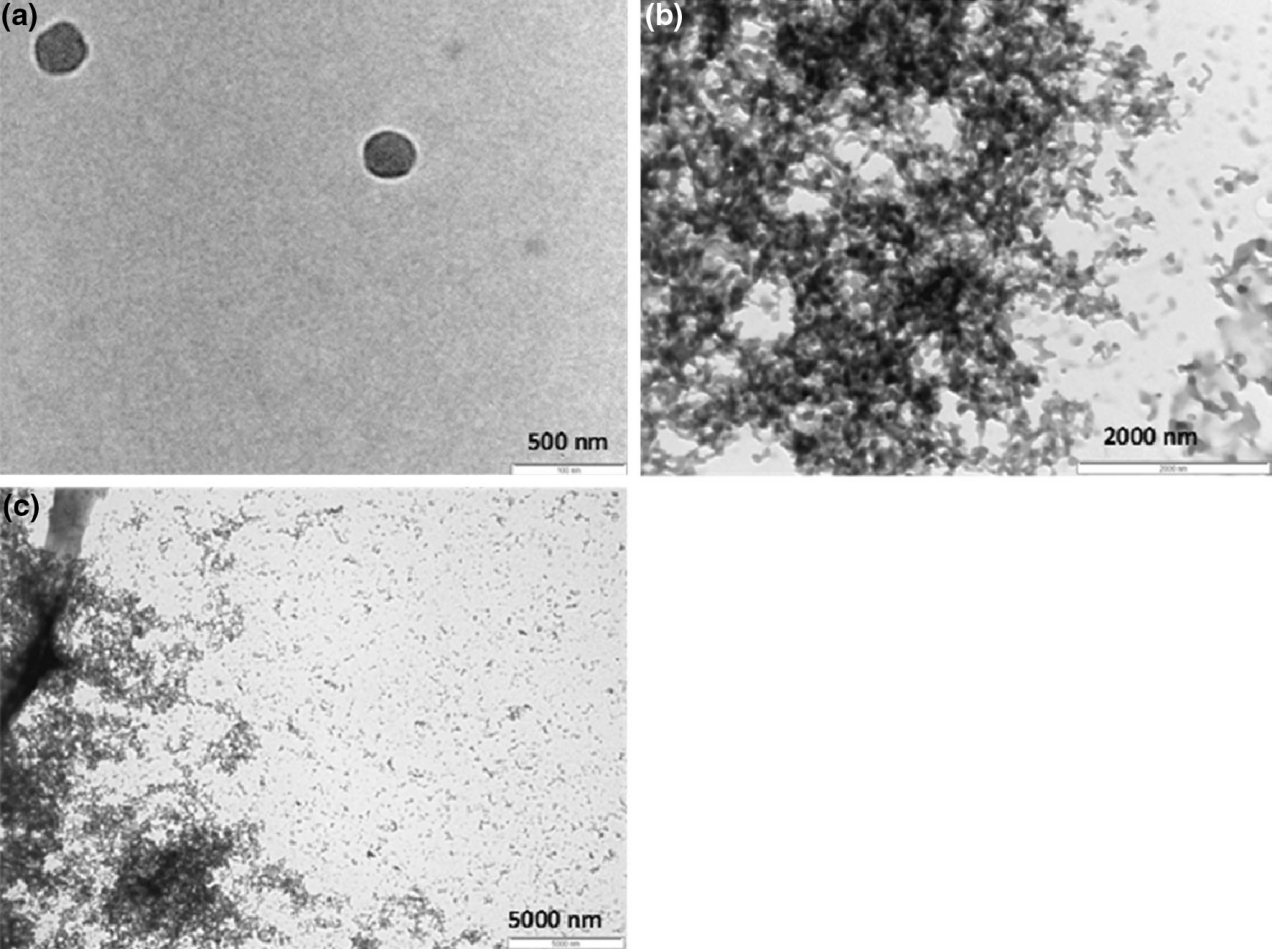
Transmission electron micrographs of CHX–HMP NPs showing single nanoparticles and larger aggregates

**Fig. 2:**
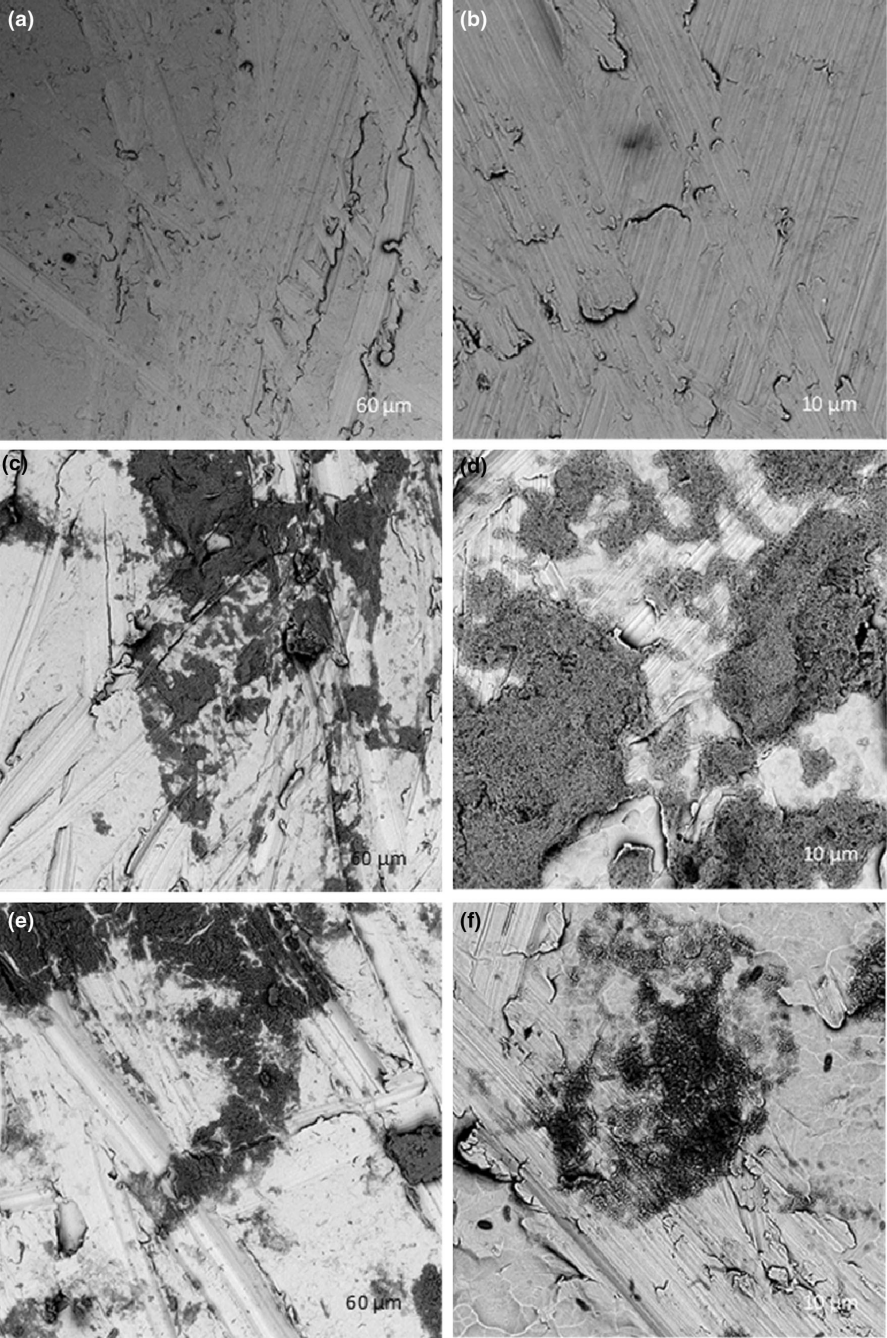
Scanning electron micrographs of uncoated titanium (**a**, **b**), titanium after CHX–HMP NP deposition (**c**, **d**), and titanium after CHX–HMP NP deposition and 95 days’ immersion in deionised water (**e**, **f**)

Large nanoparticulate aggregates, smaller clusters and single nanoparticles were observed using TEM (Fig. 1). EDX data indicated the presence of P, Cl and Na in areas dense in NPs, as well as Cu and Au which are attributed to the TEM grids used as a substrate. Mean NP size was 49.0 nm (SD 13.1 nm) from TEM (n = 62 NPs). Porous nanoparticle deposits were observed on roughly polised titanium surfaces (Fig. 2); similar deposits were seen using atomic force microscopy (data not shown). Surface deposit was still observed after samples were immersed In deionised water for 95 days (Fig. 2).

### CHX elution from CHX–HMP NP coated titanium

**Fig. 3:**
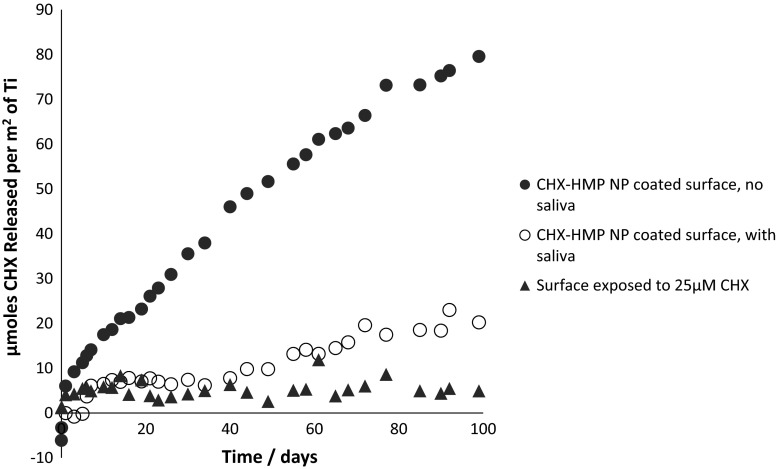
Release of aqueous CHX from NP-coated titanium surfaces, with and without a salivary pellicle, compared with control samples

Soluble CHX was released from CHX–HMP NP-coated titanium continually throughout the experimental period (Fig. 3). Approximately 4× more CHX was released from specimens that were not coated with a salivary pellicle compared to pellicle-coated specimens. No sustained CHX release was observed from specimens which had been immersed in a 25 µM CHX solution.

### Antimicrobial efficacy against *Streptococcus gordonii*

**Fig. 4:**
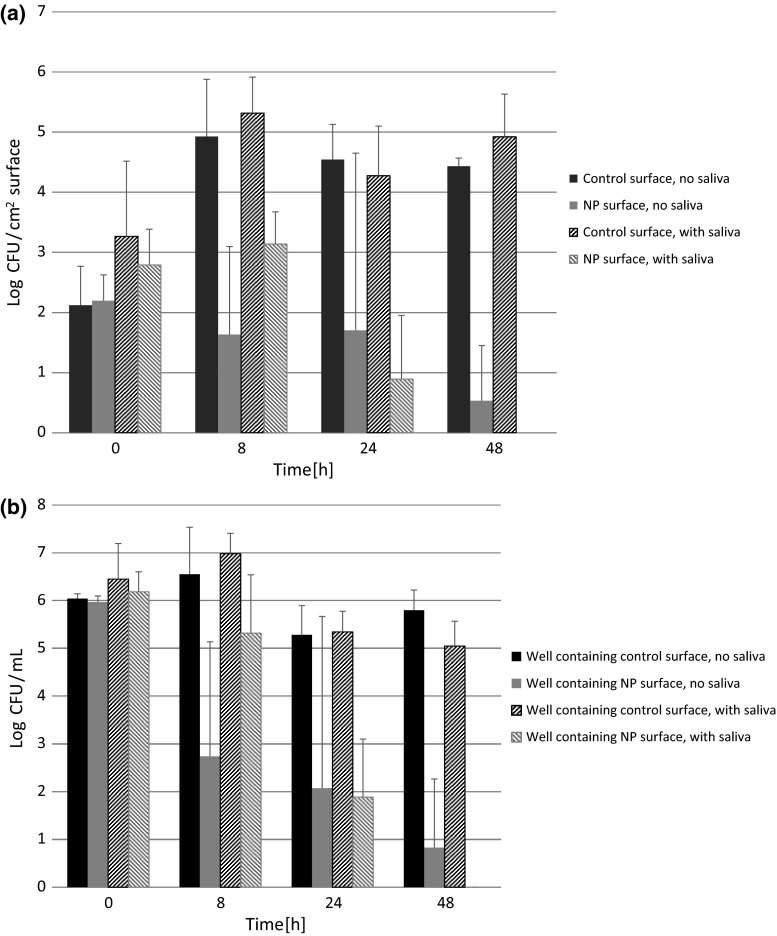
*S. gordonii* CFUs (**a**) on titanium surfaces and (**b**) in wells containing titanium surfaces, as a function of time, with error bars indicating standard deviations

The average number of bacteria present in the bacterial stock solution added to each well was 1.94 × 10^6^ CFU/mL. *S. gordonii* CFUs as a function of time on titanium surfaces and in the wells containing the titanium surfaces are shown in Fig. 4. *S. gordonii* CFUs on CHX–HMP NP-coated titanium without a salivary pellicle decreased as a function of time, whereas CFUs on the uncoated titanium increased 10^3^-fold during the first 8 h and then remained constant. There was no statistically significant difference between the surfaces at time 0 h (*P* = 1.000); the difference between the surfaces was first statistically significant at time point 8 h (*P* = 0.01). CFUs in the wells containing the CHX–HMP NP-coated titanium decreased as a function of time, whereas CFUs in the uncoated substrate remained constant. Wells containing no titanium substrate exhibited very similar CFUs to those containing uncoated titanium (data not shown). There was no statistically significant difference between CFUs in the well at time 0 h (*P* = 1.000); the difference between the wells was first statistically significant at time point 24 h (*P* = 0.001).

In the presence of a salivary pellicle (pellicle applied after the NP coating but prior to exposure to the bacteria), the results were broadly similar. CFUs on CHX–HMP NP-coated titanium decreased over the experimental period and were reduced to zero by 48 h whereas CFUs on the uncoated titanium increased 100-fold in the first 8 h then remained constant. There was no statistically significant difference between the surfaces at time 0 h (*P* = 0.979); the difference between the surfaces was first statistically significant at time point 8 h (*P* = 0.002). CFUs in the wells containing the CHX–HMP NP-coated titanium decreased with time, reaching zero by 48 h, whereas CFUs in the uncoated substrate and blank wells remained constant. Wells containing no titanium substrate exhibited very similar CFUs to those containing uncoated titanium (data not shown). There was no statistically significant difference between CFUs in the well at time 0 h (*P* = 1.000); the difference between the wells was first statistically significant at time point 24 h (*P* = 0.012).

### Live/dead imaging

**Fig. 5:**
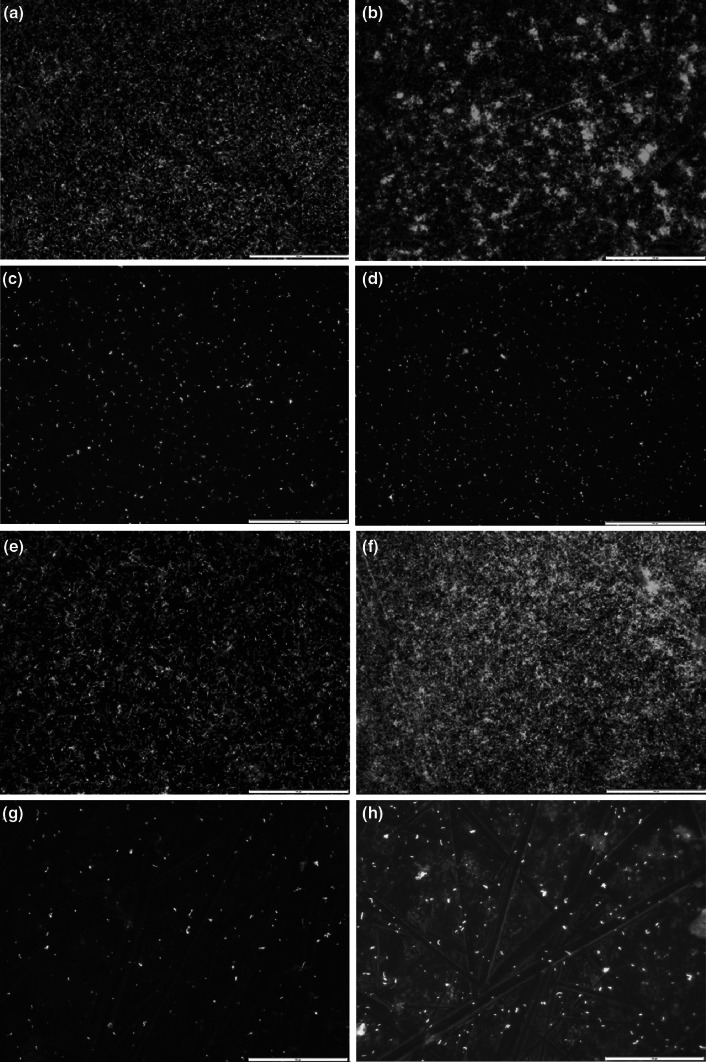
Optical micrographs showing titanium surfaces exposed to a suspension of *S. gordonii* in growth medium where *S. gordonii* is fluorescently labelled as alive (*green*) or dead (*red*). Uncoated titanium, 24 h (**a**) and 48 h (**b**) exposure; CHX–HMP NP coated titanium, 24 h (**c**) and 48 h (**d**) exposure. Uncoated titanium *coated with salivary pellicle*, 24 h (**e**) and 48 h (**f**) exposure; CHX–HMP NP coated titanium *coated with salivary pellicle*, 24 h (**g**) and 48 h (**h**) exposure. *Scale bar* is 100 µm (Color figure online)

There were more live bacteria on uncoated titanium substrates than on NP-coated titanium after 24 and 48 h (Fig. 5). This was more pronounced in the presence of an acquired pellicle, where no bacteria were observed on the NP-coated titanium or in the surrounding media after 48 h.

## Discussion

NPs with an average size of 49.0 nm (SD: 13.1 nm) composed of CHX and HMP (indicated by the presence of Cl and P) formed aggregated NP clusters (Fig. 1). The presence of Na in the EDX spectrum suggests the persistence of Na from the initial sodium HMP solution, most likely an artefact of drying the whole NP suspension onto the TEM grid, which contains all reaction by-products. Another reason for this could be the electrostatic attraction between the aqueous Na^+^ ions and the negatively charged NPs, which have surface charge −50 mV [23].

NP aggregates adhered to titanium specimens immersed in the NP suspension forming porous micron-sized deposits surrounded by large regions of what appeared to be bare titanium (Fig. 2). This rapid attachment to the titanium surface is postulated to be due to electrostatic attraction resulting from the highly charged nature of the particles; this is further supported by the reported deposition of the same nanoparticles onto a variety of surfaces including glass and ethylene vinyl acetate (EVA), a widely used biomedical polymer [22, 23]. Nanoparticle surface deposits were observed on titanium surfaces after 95 days immersion in deionised water (Fig. 2), but there were fewer of them compared with freshly coated specimens, which is thought to be because a proportion of the coating had dissolved releasing the soluble CHX.

A sustained release of CHX was observed from CHX–HMP NP-coated titanium without pellicle (Fig. 3). This compares favourably with other methods to functionalise materials with CHX, such as a component of a polybenzyl acrylate [24] or microporous silica [25] coating where the CHX release decays rapidly over the first hours or days. CHX was also released by specimens coated with CHX–HMP NPs and an overlaid salivary pellicle, at a lower rate and with larger variance in the elution data. It is thought that the passage of CHX through the pellicle is inhibited owing to the ability of the salivary pellicle to act as an ion-permeable network, inhibiting the diffusion of ions through it [26]. It is possible that CHX ions are released from the NPs but remain localised at the titanium surface and/or within the pellicle film, which would explain the difference in antimicrobial activity observed in the presence of a pellicle.

At t_0_, there was no significant difference between CFUs of *S. gordonii* on specimens coated with CHX–HMP NP and uncoated surfaces, with and without a salivary pellicle. More bacteria adhered in the presence of a salivary pellicle, indicating the mediation of bacterial adhesion afforded by the proteinaceous film (Fig. 4). After 8 h, CFUs on the uncoated titanium had increased 1000-fold in the absence of a pellicle and 100-fold in its presence; the final CFU reached at 48 h was similar with and without the pellicle. CFUs remained stable for the NP-coated titanium but increased for uncoated titanium, both with and without a pellicle, resulting in a significant difference between the NP-coated and uncoated substrates across all time points except t_0_. In the presence of a pellicle the CFUs on NP-coated surfaces decreased significantly after 24 h, before falling again to zero for all specimens. A similar pattern was seen for the bacteria in the surrounding growth medium; these were reduced in those wells containing NP-coated specimens but not for those containing uncoated titanium. The fact that wells containing uncoated titanium and empty wells exhibited very similar numbers of bacteria indicates that the titanium per se did not affect bacterial growth in the medium. The main difference afforded by the pellicle was that viable *S. gordonii* were reduced in the absence of a pellicle but eliminated in the presence of a pellicle, both on surfaces and in the medium, which suggests that active CHX was sequestered in the pellicle layer and thus offered a more concentrated source of antimicrobial for these specimens.

These observations are largely corroborated by the findings of the optical microscopy using a live/dead stain. A reduction in live bacteria was observed on the NP-coated surfaces after 24 and 48 h, compared with uncoated surfaces (Fig. 5). The presence of some live bacteria on the NP-coated surfaces after 48 h, when no CFUs could be retrieved, suggests that the CHX NPs were in this case offering a bacteriostatic rather than bactericidal effect. The small, but statistically significant, reduction in CFU number for the uncoated and blank wells after 24 and 48 h is hypothesised to be due to the reduction of nutrients in the media caused by the metabolic action of the bacteria.

Since twice-daily CHX application in situ reduced plaque formation on titanium abutments in the mouth [27], a means to apply CHX for a sustained period without regular intervention, and/or for those areas which are not accessible to a mouth rinse or other product, could have a beneficial effect with regard to biofilm formation. A randomized multi-centre clinical trial indicated that multiple applications of chlorhexidine chips over 18 weeks resulted in substantial improvement in sites with peri-implantitis, indicating that a local environment with a sustained presence of chlorhexidine was clinically beneficial in infected sites [28].

The antimicrobial efficacy demonstrated here can be expected to be sustained only as long as there remain CHX–HMP NPs to deliver soluble CHX. Since the release mechanism relies on dissolution, this will be inherently limited by the maximum NP coverage that can be applied while still allowing effective osteoblast colonisation and maturation and production of bone. The dissipation of CHX from the implant site is likely to be slower in vivo than in the experimental model reported here where the specimens are immersed in water and vigorously agitated. Irrespective, since the primary risk period for colonisation of the implant surface with microbes is during or soon after implant placement [9], and CHX offers effective treatment of peri-implant mucositis [29], a coating which could deliver CHX for weeks or months after surgery may be of utility even if the effect is not indefinite.

## Conclusions

CHX–HMP NPs were used to create a porous aggregating coating on titanium surfaces. This coating released soluble CHX continually over the duration of the study. Growth of *S. gordonii* was reduced on the NP-coated surfaces compared to uncoated titanium and titanium exposed to an aqueous solution of CHX. With further optimisation, this technology may find application in the prevention and/or treatment of peri-implant infection.
